# Recommendations Regarding COVID19 Infection in Rheumatic Patients in Greece

**DOI:** 10.31138/mjr.31.1.6

**Published:** 2020-03-31

**Authors:** The Greek Rheumatology Society and Professional Association of Rheumatologists (ERE-EPERE)

**Keywords:** COVID19, recommendations, rheumatic diseases, immunosuppressives, biologic agents

## INTRODUCTION

The Greek Rheumatology Society & Professional Association of Rheumatologists (ERE-EPERE) in response to the recent COVID19 pandemic has issued the following recommendations regarding COVID19 infection in patients with rheumatic diseases.

## ARE PATIENTS WITH RHEUMATIC DISEASES AT INCREASED RISK FOR SEVERE DISEASE (“VULNERABLE POPULATION”)?

The majority of patients with COVID19 infection (80%) in the general population develop mild to moderate disease (including those with or without pneumonia), whereas ~14% severe (severe pneumonia with hypoxemia and lung infiltrates) and ~6% critical (respiratory failure, septic shock and/or multi-organ failure) disease.^1^

According to the published reports so far, older age appears to be the most important risk factor for severe disease.^2–4^ Other risk factors include cardiovascular and respiratory diseases, hypertension, diabetes mellitus and probably male sex.^2–4^

Until today, no data are available regarding the risk for severe disease in patients with auto-immune/auto-inflammatory rheumatic diseases who are receiving or not immunosuppressive or immunomodulatory agents.^2–4^ There has been only one report of 18 patients with history of cancer from China, which showed that their risk for developing critical disease or death was higher (39% vs. 8%) compared to the rest of the infected population (n=1.572).^5^ However, it should be noted that these patients with history of neoplasia were older (63 vs*.* 49 years old) and more frequently smokers (22% vs*.* 7%).^5^

It is unknown whether the lack of data for rheumatic patients is due to the low frequency of autoimmune inflammatory diseases in the general population (~2–2.5%), especially those treated with high-potency immunosuppressives, to inadequate recording, or to an actual absence of increased risk. However, the lack of published data for these patients does not exclude the possibility that they are at higher risk for a severe outcome.

Based on the above limited data, our National Society, taking into account the critical situation with the COVID19 infection worldwide and the potential risk for severe disease in our patients:

- *Recommends that patients with autoimmune/auto-inflammatory rheumatic diseases could be classified as being at higher risk for severe infection, based on their:*
  - underlying rheumatic disease (eg, vital system involvement such as: respiratory, cardiovascular, renal, hematologic, etc.)
  - immunosuppressive/immunomodulatory treatment and
  - specific co-morbidities (old age, hypertension, cardiovascular or respiratory diseases, diabetes mellitus)
- *Urges Rheumatologists to state clearly in their medical reports for rheumatic patients who are still employed, their:*
  - type of rheumatic disease,
  - immunosuppressive/immunomodulatory treatment administered,
  - characterization as a “vulnerable group” (based on Recommendation A), and
  - need for increased protective measures (leave of absence, change of working post, working from home, etc., depending on the nature of their employment), taking always into account their exposure risk to the virus.

## WHAT IS THE IMPACT OF ANTI-RHEUMATIC THERAPIES ON THE RISK FOR SEVERE COVID19 INFECTION?

Regarding the group of patients who receive immunomodulatory or immunosuppressive therapies, particularly biologics, there are inadequate data as of today, regarding their effect on the outcome of the infection (mild/moderate *vs.* severe/critical).

Although the World Health Organisation (WHO),^6^ the National Organisation of Public Health in Greece (https://eody.gov.gr/covid-19-odigies-therapeias/) and experts in the field^7^ do not recommend glucocorticoids (GCs) in patients with severe COVID19 infection, in a recent retrospective study from China, administration of methylprednisolone in patients with acute respiratory distress syndrome (ARDS) resulted in a significant reduction in mortality (from 62% to 46%, Hazard Ratio = 0.38).^8^ Randomized trials currently in progress (data from: https://clinicaltrials.gov) are expected to answer this specific question.

In addition to GCs, a number of anti-rheumatic drugs (hydroxychloroquine, tocilizumab, Intravenous Immunoglobulin) are also being tested for their potential usefulness in patients at different stages of COVID19 infection (data from: https://clinicaltrials.gov). In a recent study, a Janus kinase inhibitor (Baricitinib) was suggested as a potential therapy based on artificial intelligence data.^9^

## WHAT SHOULD PATIENTS WITH RHEUMATIC DISEASES WHO RECEIVE ANTI-RHEUMATIC TREATMENT DO?

As of today, International Rheumatology Societies^10 –12^ do not recommend discontinuation of anti-rheumatic therapies in rheumatic patients who are asymptomatic or have not been clearly exposed to infected patients.

Based on the available data, ERE-EPERE:

- Does not recommend the discontinuation of immunosuppressive/immunomodulatory therapies (especially biologics) in all rheumatic patients
  The decision for temporary discontinuation (per os, subcutaneous treatments), postponement (intravenous treatments) or switching (from intravenous to subcutaneous) treatment should be individualized by the treating rheumatologist based on the underlying rheumatic disease (type, severity), the type of immunosuppressive/immunomodulatory treatment, the co-morbidities (old age, hypertension, concomitant cardiovascular or respiratory problems, diabetes mellitus) and the exposure risk to the virus of the individual patient.
- *Recommends the immediate discontinuation of immunosuppressive/immunomodulatory therapies (with the exception of GCs and hydroxychloroquine) in patients with rheumatic diseases who:*
  - had high risk contact (duration> 15 min, distance <2 m) with a patient with confirmed COVID19 infection, or
  - have symptoms of acute respiratory infection or
  - develop COVID19 infection

The above recommendations may be revised based on the emerging new data and the new guidelines issued by our National Organization of Public Health.
